# Changes of intuition in paranoid personality disorder

**DOI:** 10.3389/fpsyt.2023.1307629

**Published:** 2024-01-10

**Authors:** Kerrin Artemis Jacobs

**Affiliations:** ^1^Department of Philosophy, Ethics, and Religious Studies, University of Hokkaido, Sapporo, Japan; ^2^Center for Human Nature, Artificial Intelligence, and Neuroscience (CHAIN), University of Hokkaido, Sapporo, Japan

**Keywords:** paranoia, personality disorder, oikeiôsis, intuition, predictiveness

## Abstract

Wherever psychopathology operates with the concept of (disorders of) the self and personality, it can address the role of the intuitive access we have toward ourselves, others, and the world. This study discusses the concept of *oikeiôsis*. It examines its role in explaining paranoia as a change in *intuitive* self-and-world relatedness. In the first *step*, symptomatic features of paranoid personality disorder are sketched, with a focus on the explanatory role of attentional and interpretative biases, which correlate with significant changes in intuitive processing. In the second *step*, the prototypical phenomenality of feelings of unfamiliarity and mistrust are discussed against the backdrop of changes of oikeiôsis in paranoid personality disorder. In the *third step*, the main therapeutic challenge in treating paranoid personality disorder—building a trustful relationship—is explored. It is concluded that the notion of oikeiôsis resonates particularly with introspection-based therapeutic approaches.

## 1 Paranoia and paranoid personality disorder

Paranoia is a symptom found in several psychopathological conditions. The spectrum of paranoid reactions has been suggested to build a continuum (1–3) reaching from neurotic forms (F23.2) of a paranoid tendency, which are held not to be uncommon in the general population (4, 5), to paranoid personality disorder [in the following: PPD; classified under *Cluster A* together with schizoid and schizotypal in the DSM; ((6), p. 399–426)] and to more severe forms in psychotic manifestations (7), e.g., in paranoid-hallucinatory schizophrenia or its specific expression in older age or “contact deprivation paranoia” (8) for which persecutory delusions are seen as forming the end of the assumed continuum (9). The DSM-V [5th ed.; DSM−5; (10)] outlines primary diagnostic criteria for paranoia, focusing on a general mistrust and suspicion of others' motives (*criterion a*). This is further detailed through seven specific sub-features: (1) believing that other people are intentionally threatening or acting in a harmful way; (2) pervasive doubts about the trustworthiness/loyalty of others; (3) avoidance of confiding in anticipation of becoming betrayed; (4) misinterpreting ambiguous remarks as intentionally hurtful; (5) holding grudges against others; (6) believing that others are assailing one's character, which can cause vindictive reactions; and (7) a tendency in romantic relations to suspect partners as being unfaithful. Four of these criteria must be present in addition to *criterion b*, which requires that these symptoms cannot be attributed to a psychotic episode, bipolar disorder, or major depressive disorder with psychotic symptoms. These features are already expressions of symptomatic changes of self-and-world relatedness: the world is tendentially perceived as a dangerous place, and there is a sensitivity to rejection or the fear of *social exclusion* (11) that comes in tandem with recurrent suspicions and quarrelsome insistence on one's own rights, as well as the development of unfounded beliefs that appear as doxastic in nature and typically center around others as deliberatively intending to cause harm [for a review of the different definitions of the content of persecutory delusions, see ((12), p. 408ff, 412)]. Often, attempts to persuade people with paranoid personality disorder remain mostly unsuccessful and can be seen as rather counterproductive interventions since they can reinforce the paranoid person's mistrust, which has also been discussed as one of the main challenges of therapeutic treatment and a reason for general disinclination to seek treatment (13, 14). Personality disorder is diagnosed when a person's thoughts show an impairment in *reality testing* [orig. cf. (15)], which is not exclusively observed in cases of psychotic forms of paranoia and also in PPD. The severity of paranoid delusions seems to correlate with the intensity of affectivity, such as fear and aggressive moods, which may potentially lead to violent behavior [e.g. (16, 17)]. Albeit coming with an increased risk for violence, especially in comorbidity with other disorders (such as antisocial personality disorder), this must not necessarily be the case in PPD. Self-defeating thoughts, social withdrawal tendencies such as avoiding (further) conflicts or anticipated harms, a proclivity to be less open to changes and to let go of rigid cognitive patterns, being hypersensitive (to rejection or authority), and ruminating about what is (intuitively) sensed as hostile and threatening are factors that play a significant role in PPD. Moreover, a correlation has been suggested between *unstable self-esteem* and PPD, which relates to the hypothesis that paranoia has a *defensive function* that is expressed, e.g., in the symptomatic blaming of others for the occurrence of negative life circumstances and particular events [as Bentall et al. (7) suggests] and in querulous behavior, which “should once more take its place among the legitimate concerns of mental health professionals” (18). Alternatively, paranoia has been seen as reflecting a *self-esteem problem* as people with lower self-esteem range higher in paranoid beliefs than non-paranoid controls [e.g., (19)]. The severity of symptoms as *ego-syntonic* or *ego-dystonic* experience is reflected in the “*poor-me*” vs. “*guilty-me*” debate on paranoia, e.g., with respect to either perceiving oneself as a victim and projecting the respective negative feelings onto the perceived predators or suffering from feelings of shame and guilt as forms of internalizations of anticipated negative reactions from others [e.g., (20) *ibid*, p. 41; (21, 22)]. These results confirm developmental psychological studies that state a correlation between (insecure) attachment styles and paranoia [e.g., (23)] and those studies that stress the role of developmental trauma for the onset of paranoia as a paradigmatic symptom of self-disorders (24, 25). In psychotic conditions, in contrast, the self-relation can change even more drastically in terms of mistrust in one's own perception and embodiment, for instance, in directions of extreme derealization and depersonalization experiences that have been described in the phenomenology of psychiatry as “disembodiment”, and refer to severe distortions of trust in the most common, rather unquestioned basic assumptions of our daily life (such as that one has a body, or that dead people cannot be alive, or that the people in one's family most likely have not been replaced by actors) [see exemplarily: Fuchs (26), Fuchs (27), Fuchs (28), Fuchs (29), and the discussion in Ratcliffe (30) of *Cotard* delusion p. 155–180 and *Capgras* delusion p. 139–63].

In PPD, actions often have an almost delusional or compulsive nature. Some actions are felt as unavoidable to ward off anticipated dangers [e.g., obsessively protocolling, searching for evidence of persecution in the environment, and performing rituals for protection from others, as in spiritually induced or religious paranoia; see: Jacobs (31), Ash (32), Bhavsar and Bhugra (33), Pfaff et al. (34)]. Paranoia apparently includes *ideas of reference* (35), which means that there is an “appropriation” of things, narratives, events, and persons that become imbued with a particular meaning, ascribed a specific role, and integrated within the individual delusional belief system accordingly. The respective beliefs and corresponding emotions, desires, and actions “make sense” within the paranoid reality but become questioned because of a lack of an intersubjective justifiable, i.e., rational basis (although it must be reminded that just because a belief is not seen as intersubjectively rationally justifiable, there is a chance that it might turn out not be based on false presuppositions). Particularly in psychotic forms of paranoia, severe doubts in *certainties* (36) or “bedrock beliefs” (37, 38) of our daily encounters appear. These fundamental doubts have been described by Karl Jaspers as accompanied by “delusional moods” [germ. *Wahnstimmungen*, cf. (39)] in which the experiential possibility of feelings of safety and trustful belonging gets lost and is replaced with an ominous feeling “that something strange is going on”. Being in constant worry of threats and harm apparently reduces the possibility of sharing an experiential horizon with others (at least with those that do not share the same paranoid content). The intersubjective encounters are overshadowed by the atmosphere of suspiciousness, feelings of alienation or being socially excluded, or even the target of non-trivial harmful set-ups and plots, according to which PDD comes with the experience of a lack of social recognition or feelings of being victimized by reification. The assessment of the perceived severity of threats, obstacles, and irritations builds a continuum that has become addressed with the so-called “paranoid hierarchy” [cf. (40), Figure 1].

Other processes that have been thoroughly marked as associated with the onset and maintenance of paranoid beliefs [for an overview, see: cf. (41, 42)] are *negative affectivity* [such as depressive affect e.g., Freeman et al., ibid. (43); in psychosis see: Hartley et al. (44)], a higher level of *dysfunctional emotional regulation* for which avoidance has been paradigmatically stressed [c.f. (45)], and *altered cognitive schemas* due to *attentional* and *interpretative* biases. The misinterpretations of others and situations lead to unfortunate cycling dynamics of altered perception, misinterpretation, and enaction. It might be useful to put stress on perceptual and pre-reflexive cognitive and affective biases, particularly in relation to higher-cognitive “self-reflexive” processing, to address the distortions of trust and belonging as structural changes of the evaluative dynamics between pre-reflexive and (self-) reflexive dimensions of consciousness. These biases are the structural prerequisites for transforming the world into a hostile or unhomely place and/or predicting that other people are potentially harmful. Generally, a bias will be stronger when the information being processed has more direct relevance to the disorder and its symptoms. Thus, although a lot of disorders (such as depression) can be explained by attention and interpretation biases *favoring negative information in general* (46–48), information that allows for more specific paranoid interpretations would be expected to elicit the strongest biases in a paranoid population [cf. (49), p. 4]. Biases, then, should be closely associated with interpretations that reflect a threat of harm to the paranoid self, such as the suspicion that strangers chatting nearby are talking badly about one or the suspicion that a stranger's stare is intrusive or mean. Evidence suggests that persecutory delusions are maintained by biases particularly in the interpretation of emotional *ambiguity* (50). These attentional and interpretative biases can explain how the world is “made a match”, as these prime agents focus on their negative outcomes or misinterpreted social interactions, which then builds the experiential basis for getting (re-)enacted, according to which the biases become even more rigid [for a conceptual view of the structural rigidity of evaluative pattern formation, see: cf. Jacobs ((51), p. 6ff)].

From a phenomenological perspective, the formation of rigid attentional and interpretative biases indicates an inability to stay experientially open to different forms of self-actualization. It seems also typical for PPD that certain “immunization strategies” become activated: A classic is a phenomenon of “double bookkeeping” [originally coined by Bleuler (52)]; see also: Porcher (53); Stephensen et al. (54)], which was initially described as a protective strategy in schizophrenic patients but seems to also be a defense mechanism in PPD. Paranoid people can indeed register and “cope” with the possible experience of evaluative inconsistencies between their doxastic beliefs on the one hand and the (rational coherence) constraints of certain environments on the other hand. If the double bookkeeping, i.e., the holding on to the delusional convictions while simultaneously (trying to) adjust to what is required from one given certain situational necessities, can be sustained, then the inconsistencies between personal beliefs and common sense and between inner reality and the imperative of an external “objective” reality might momentarily become bridged. This serves the function of sustaining self-coherence and to avoid social conflicts. On the contrary, the discrepancy between the paranoid assessment of a situation and those who doubt, criticize, or try to persuade paranoid people can cause a great amount of stress, unease, and insecurity, partially because there might be glimpses of mistrust in one's own perception and interpretation, or fears of being socially shamed for it, or, in contrast, it creates emotional reactions such as anger and vindictiveness because critique is seen as a direct attack to one's self-worth and personal integrity. With respect to this “clash of realities”, it must be considered that most people who have paranoid symptoms often have experiences of being stigmatized by others (i.e., due to harsh reactions, such as “you are a helpless paranoiac”, “it will be a false alert as always”, and “I better ignore this” etc.), which unfortunately fuels the biased view, as paranoid people see their (real) experiences being invalidated by others. The hindrances to fully *reason together* reflect what Karl Jaspers described as the challenge of a general *limitation of understanding* (germ. *Verstehensgrenze*) of psychotic content [(55), p. 98], which he also elaborated in limits of therapeutic understanding [(56), p. 11]. Karl Jaspers has described the “risks of a fracture of synchronic unity or self-coherence” for the case of psychosis in the following way: “[The patient's] world has changed to the extent that a changed knowledge of reality so that any correction would *mean a collapse of being itself* , in so far as it is for him his actual awareness of existence. Man cannot believe something that negates his existence” [Jaspers (56) p. 105, italics KJA]. This means that poking at that inconsistency of individual belief and external facts (counterproofs) to expose someone or “reveal” delusion should not be underestimated as an exacerbation factor and as a source for aggressive reactions also in PPD.

In the following, paranoid experiences are sketched with respect to changes in *intuitive* processes, which relates the clinical symptomatic to a philosophical phenomenological view on the loss of trust and familiarity in PPD.

## 2 Changes of intuition in paranoid personality disorder

### 2.1 Oikeiôsis

The concept of oikeiôsis (oκεíω*σις*) has been fragmentally handed down [of import are passages in Diogenes *Vit. Phil*. 7.85; cf. David (57) and Cicero *Fin. 5.42* (58, 59)]. Its etymology is rooted in the word *oikos* (oκoς), which is the word for household, house, or family. “*Oik*-“ can also mean “what belongs to me”, while “-*eiôsis*” refers to becoming familiar with something or what belongs to me, which is in the Stoic tradition synonymous with “*self-knowledge*”^1^. Similarly, the term *oikeiôtes* refers to the perception of something as one's own and addresses in the most general way the sense of belonging [cf. ((60, 61), p. 114–49)]. The term also invokes the sense of *being at home* or of *becoming familiar* with something [cf. (62, 63)]. An important distinction to make is the difference between *internal* and *external* oikeiôsis, as introduced by *Hierocles the Stoic*: while the former refers to the appropriation of one's own self and constitution, the latter refers to the process of *familiarization with other people* and an orientation toward external goods that are the outcomes of oikeiôsis. This also entails *appropriate behavior toward others*, which is a prescription and duty of reason, thus a crucial part of living a coherent, rational, or good life according to the stoic tradition. Oikeiôsis as an *active* way of *self-and-world-acquisition* is, in principle, a matter of extension, as it has been paradigmatically pointed out by Hierocles (64). He describes the realm of obligation and ethical concern as something that can be extended beyond the inner circle of family and peers and therein refers to a practical normative understanding of human interrelatedness in terms of what “we owe each other” as being reasonable, interdependent beings. We can reflect on our own existential situation—our oikeiôsis—as a “being-with” in such a way that processes of becoming familiar with the world are not solely centered on proper (or egotistical) self-realization but must also include a sense of reasonable relatedness to our fellow beings, mediated by and simultaneously archived through an intellectual and practical commitment to common sense. In contrast, in psychopathologies such as paranoid personality disorder, there is often a symptomatic disruption in self-and-world-appropriation as it relates to “familiarity, feelings of belonging and trustful relatedness” and, as we have seen, is associated with problems of social adjustment, which are often due to an altered perception and interpretation of life that is incongruent with common sense.

I would like to stress three *structural relata for explaining the “paranoid” turn of oikeiôsis* in relation to what has been sketched above: (1) Paranoia is the experience of the world turning into an unsafe, unhomelike, and hostile place (*world-relation*), (2) in which people are perceived as predators, threatening and are seen as no longer to be trusted (*relations to others*), and (3) paranoid people also often lose trust in their own capabilities or even their own perceptions and deal with self-esteem problems (*self-relation*). These changes can become further specified considering the question of whether PPD is either a kind of (1) *distortion* of oikeiôsis as it appears as restricting patients' experiential possibilities of “feeling at home in the world” or whether (2) PPD is rather a variant of an “oddly reversed oikeiôsis”, i.e., a “pathology of normalcy”, although this may be assessed as severe maladjustment. If we accept that one can situate perfectly in a full-blown paranoid reality, which allows for the inclusion of feelings of “familiarity” (even if it is “externally” assessed as even clinically relevant, non-trivially harmful dysfunctional state), then this apparently differs from the idea of paranoia *as a fracture* of *oikeiôtic* processes. What seems to speak for PPD as a direct opposite of “normal” oikeiôsis is that paranoia shows *ex negativo* what we normally take for granted but what seems more or less absent in paranoid experience: that we are not “at war” with the world and others, we do not fundamentally distrust the practical certainty of live-worlds presuppositions, we rather assume that other people are able and willing to participate in adequate mutual social recognition relations, and we rather try (as difficult as this might be) to adapt by “making the world our oyster” in spite of all the terrible things that go on in the world. Alternatively, PPD could be seen as a specific form of oikeiôsis that pays more credit to unique self-and-world appropriation against all odds of perceived obstacles, persecution, and social pain. Emil Kraepelin once “call[ed] insanity the chronic development of a permanent delusional system with perfect preservation of prudence” [cf. Emil Kraepelin (65), 1713, transl. KJA]. Paranoid oikeiôsis might include (in psychotic conditions) even a kind of “un-botheredness” due to the diminished capacities of illness insight and reduced affective resonance, while in the condition of PPD, being able to be bothered (by others) and trying to insist on one's point speak of an intact agential capacity for effective self-realization, albeit it can be assessed as (even as a clinically relevant) form of maladjustment, or is individually experienced as stressful. Indeed, there are good examples of this “persistence”: this phenomenon of actively situating in a “stable” paranoid reality has been exemplarily documented in the first-person patient narrative of Friedrich Krauß (1791–1868) known as the “*Nothschrei eines Magnetisch-Vergifteten*” [*A Cry of Distress by a Victim of Magnetic Poisoning* Krauß (66)]. This thousand-page self-report is the most comprehensive 19th-century description of a (episodically) psychotic, paranoid experience that centers on Krauß' denouncing a Dutch family and their helpers of over 40 years for wanting to kill him through “magnetic” influences, whom he accused of maltreating his body from afar by means of a “magnetic fluid”. Krauß reported that he had been mistreated daily and that this family wanted to murder him, control his body, read his thoughts, invade his dreams, and follow him with omnipresent voices commenting on him. It is easy to recognize how spiritual delusions and hallucinations seem strongly influenced by contemporary events, socio-demographic factors, and the particular culture, as the mesmeric background of the delusional beliefs show [i.e., Franz Anton Mesmer's (1734–1815) theory of “animal magnetism”, see: Brückner (67), Brückner and Jádi (68)], but beyond that, it illustrates a “counter-existence” as Krauß fought his “enemies” in public for decades, pointing to a desire *for* belonging, familiarity, and trust, namely, in one's own “truth” and resistance.

Albeit speaking of “distortions of oikeiôsis” might be closer to the originally intended meaning of the notion, as it already presupposes a theory of “objective” goods and harms, and that of (in)adequate enaction, according to which “having a paranoid personality” could be assessed as harmful dysfunctional condition, the second reading would not automatically incline one to conclude that there is a “disorder” involved, particularly not with respect to the determining criterion of self-and-world appropriation. People with PPD apparently get engaged with the world, extend their relatedness (e.g., in terms of ideas of reference), and often are convinced that they (instead of others) are on the right track. A combination of both variants may come even closer to the lived reality of PDD: there might be aspects of one's daily encounters where people with PPD adjust and experience themselves in meaningful relations that might even allow for feelings of trust, belonging, and familiarity, and those dimensions in which exactly these are restricted by the biased attention and interpretation of self, others, and the world. This is not a paradox if we adopt a more *situation-specific view* on *intuitive* self-and-world appropriation, according to which it might be possible that severe distortions of trustful relatedness can come together with phenomena of trust in one's beliefs and the reliability of one's own perceptions.

### 2.2 Intuition

The concept of intuition, present throughout, has undergone various interpretations but is generally discussed for its central role in human cognition. What is at stake is an *immediate grasp* (e.g., of first principles or concepts), which in the historical-philosophical view suggests intuitions to be a special mode of cognition distinct from both perception and inferential reasoning. Another aspect of intuitive cognition can be highlighted in Spinoza's conception of a *scientia intuitiva* (69, 70) or Kant's reflections on the Transcendental Theory (Part II) on the sources of knowledge (71, 72), which ties it up to “gut feelings”. The latter refers to an *appraisal capacity*, i.e., having implicit or non-propositional knowledge, which is essential for large parts of human life and is thought of as a necessary component of all cognitive processes. Thus, for example, in phenomenology from Husserl [e.g., as elaborated by Levinas (64)] to Schmitz (73), “having an intuition” is more than a mere registering of facts, but a mode and at the same time an expression of a pre-reflexive grasping beyond the purely factual elements of a situation. Intuitions, after all, are also not infrequently seen as “proof” for certain statements and vice versa: when something appears as counterintuitive, we refer to the moment of frowning, i.e., when what is true runs counter to some sort of basic assumption, and therefore appears as paradoxical. Most people listen to their gut, would probably have a feeling for one's intuitive sense, and would eventually also differentiate among several scenarios where relying on gut feelings seems (in)appropriate. Intuiting moreover reveals to us what we individually spontaneously evaluate as an (in)possibility and can provoke us to reflect on why exactly we have this hunch and do not feel another way, i.e., it also implies a kind of self-knowledge about one's predictive capabilities and may reveal some biases in intuiting after the truth of a situation comes to light.

Intuition is a *primordial* (rather than “sixth”) sense of self, others, and the world. There is nothing that is mystical, esoteric, or exceptional about it. Instead, it is basic “sense-making”. Intuitive information processing takes place “at the fringe of human consciousness” [Zander et al. (74), p. 3; the authors refer to the works of Mangan (75); Norman et al. (76), Norman and Price, (77)] and often has been explained in distinction to decision-making processing [e.g., (78)]. This theoretical differentiation is central to various “dual process-theories” (79) and “two-system-framework” models [e.g., (80)], according to which a difference between fast (intuitive) and slow (deliberate and analytic) thinking [e.g., (81, 82)] has been stated. Contemporary understanding, however, views these alternative information processes not as two separate systems (83), albeit those reflect different accesses for self-and-world orientation in terms of decisive enaction, as agents differentiate and consciously can decide whether they rather rely on reasoning *or* follow their “gut feelings” [e.g., (84)]. Various theories aim to define “intuition”, but many agree that it is experience-based processing that leads us to form hunches or hypotheses [cf. (85)]. The *main features* that characterize intuition and that sometimes are used to differentiate the relation between “intuition” and “insight” [cf. (74), 2–3.] are as follows: that of *non-conscious processing* and *uncontrollability*, which neither cannot be controlled or intentionally evoked [e.g., (86)], that intuitions have an *experiential* character (only in the unfolding of a situation or *ex-post*, we can falsify whether our intuition was correct), and that they have an intrinsically *motivational* dimension, i.e., people literally *feel* inclined to follow their gut (87). Intuitive processing is seen as rooted in “*tacit knowledge*” people acquire in and through their daily encounters with the world [e.g., Bowers et al. (88); for a detailed description of the *tacit system* see: Hogarth ((51), p. 191ff)], making it crucial for understanding our own self-and-world relationships, for example, the “intuitive” developing of acquaintance with something or someone ((51), p. 2ff). This is possible as the information processing in intuition takes place not in the absence of “better knowledge” but rather is a “knowing without knowing why” (89), according to which we can stress its *predictive function*: Intuitions are *appraisals* of actual and memorized experiences or thoughts. Rather than being fundamentally disconnected from higher cognitive processes, intuition is already an essential part of the ongoing *predictive dynamics* in which we situate ourselves in the world. Particularly, the *predictive processing framework* hypothesis [for an overview see: Miller et al. (90)] suggests that input (incoming sensory information and current experiences) is constantly compared against our “stored” knowledge and memories of previous experiences, which allows for prediction and to adapt to the current situation in a certain way. When a mismatch occurs (something unpredicted), the cognitive models get updated, which means there is a constant adjustment between prior models and current experience, which happens quickly, effortlessly [Hogarth ibid. (78)], automatically, and subconsciously. Intuition occurs when we have already picked up something unconsciously (“pre-reflexively”) without consciously (reflexive/self-reflexively) registering it, while the “error signals” (“mismatches”) determine “whether the model is either amended and its current hypotheses are changed to accommodate the mismatch (“passive inference”, perception), or the hypotheses are kept fixed and lead to resampling of the sensory states according to the current model (“active inference”, action)” [cf. (90), p. 798]. This might also suggest that one can even “train” one's intuition and use it in decision-making processes [e.g., Hogarth (78), p. 68–136.], and the more reliable our predictions could get, the more particular “background” knowledge (expertise) we acquire in a certain area, as this is the informative material according to which the current experience is compared against in one's intuitive grasping of the world.

My claim is that this is relevant for defining (distorted) paranoid oikeiôsis, as intuition is a necessary precondition for basic modes of relatedness. Apparently, it contributes to a pre-factual sense of *feeling at home in the world, familiar, secure, and safe*, that occurs when there is no fundamental “disruption” or “overly stressful challenges” for our predictive system. Normally, we are situated in relatively stable (e.g., not constantly dramatically changing) environments and can spontaneously transform even very “unfamiliar” (“unpredicted”) events or situations in some sort of familiarity experience exactly by “intuitive adaption”. This is possible as certain (repeating) scenarios share some prototypical features that allow us to automatically follow the “intuitive path” as we recognize significant resemblances and differences to our memorized situational blueprints or scripts. However vague and often challenging it can be in certain contexts (particularly those that require an “objective” view or intersubjectively consensual justification) to rely on one's subjective “gut feelings”, it seems that this kind of pre-factual evaluative capacity provides us with access to some kind of immediate situational understanding, often before all information to assess a specific situation in depth has been acquired. Our intuitive understanding of the world represents a primordial or pre-intentional alignment. This alignment occurs subconsciously, allowing us to synchronize with other people and our environment in an instinctive manner. The predictability of the world, others, and oneself seems to serve an *adaptive* purpose, as errors in prediction can lead us to experience non-trivial harm. Understood in this sense, intuiting provides us with some sort of sense of/about “good”, i.e., our intuitions have *salience* for us. *Nota bene*: Intuition involves higher cognitive processing because we can develop a reflexive stance toward it, which normally allows one also to register discrepancies, inconsistencies, and maybe even an awareness of one's own perceptual biases in a specific situation because what we intuit tells us a lot about how we are (subconsciously) attuned to the world. Sometimes, intuitive appraisal can even appear contradictory to beliefs or specific emotions that we might think we rationally “should” have about a particular matter. Even if we occasionally wonder why we have *this* intuition, normally, this balances out when we become familiarized with our intuition over the course of time: this means we can register our intuition and develop a reflexive stance toward it, which may allow us to become aware of our own affective and cognitive biases in a specific situation. Additionally, one can be motivated to falsify or test these gut feelings by trying to archive a better knowledge about “what is going on”. This also happens in paranoia with “harm(ful) predictiveness”, which will be explained in the following section.

### 2.3 Changes of intuition in paranoid oikeiôsis

(1) First, intuition in PPD is already the servant of pre-intentional (cognitive, affective, and conative) biases. Hunches and speculations in PPD cannot be “silenced” by a reasoning process *with or against one's own intuition*. This would imply recognizing when one is being misled (e.g., due to empirical counterproof) and being open to being corrected by others, i.e., to self-reflexively “familiarize” oneself against the backdrop of intersubjective consensually justifiable standards of rationality (or rational common sense). This assumes deliberative self-reflexive capacities, such as the ability to prioritize certain information/evaluative content, to distance oneself or objectify one's gut feelings, and, more importantly, to deal with the ambivalence that often correlates with ambiguous situations, which might require in-depth reasoning. The benefit of intuition is given with this *attunement* as appraisals help us to orientate and relate immediately, which is rather a “passive” process, i.e., runs “in the background”, but predetermines (higher-cognitive) processes that are important for agential “self-realization” in terms of rational decision-making. This means to “put intuitions in the picture where they belong”, i.e., to accept them, for instance, as indicators to be still indecisive about a certain way to act, to tolerate a momentary tension between our hunches and other evaluative states, to decide to follow our guts, etc. The crucial point in paranoid intuition is certainly about *what the biases are about*: namely, anticipated (or actually experienced) threats, dangers, and harms. Apparently, this evaluative content is hard to simply “tolerate” or rationally control in terms of being “simply” decisive by paying less attention to them or by waiting and seeing how the situation unfolds. This is equally difficult, e.g., with phobias, as you must deal with the detected threat despite knowing that “it's just a tiny spider that cannot harm you”. Paranoia is challenging, as simply suggesting that people with PPD “should better stick to reason” hardly changes the *intuitive assessment* of the subjective matter (and this seems also true for non-paranoid people). We can have irrational beliefs, desires, emotions, and/or simply find ourselves in ambivalent evaluative states (e.g., a mismatch between what is felt and what is (inter-) subjectively reasonable or rational to feel) and can be very aware of it without being automatically inclined to become “decisive” and to adjust flawlessly with what is “making the most sense” regarding common sense. The case of paranoid intuition exemplifies the “normalcy of irrationality” in one aspect, but on the other hand, differs significantly from it, as this (externally judged to be) irrational self-and-world relation does not get dissolved easily, particularly *because*—even if beliefs, emotions, desires, or actions can be assessed as hasty, unhinged, even unjustified by people with PPD—this often comes already in alliance with a (doxastic) *belief that one's intuition cannot be misled* and that it is, *therefore*, justified to hold onto a certain assessment of a situation or to behave in a particular way. The *development of interpretative biases* is central to the onset and maintenance of psychopathology [e.g., in psychosis, e.g., Yiend et al. (91)] or specific delusions [e.g., *religious* ones: White et al. (92); Pfaff et al. (35)] and *respective changes of the predictive background processing* that “feeds” and is fed by these biases through the processes of constant (re-)enaction. Central to maintaining these doxastic evaluative patterns is the fact (feels, believes, and desires) *that one's intuition about something or someone is correct* according to the vicious circle between pre-reflective and reflexive spheres; the predictions (backed by appraisal) and higher cognitive situational assessment *stabilize each other* and become installed in the ongoing enactive dynamics of paranoid oikeiôsis that then changes the relation to self, others, and the world, respectively.
(2) Second, this can be related to a more “functionalist” view of intuitive processing as serving adaptive goals. I have spoken above of adaptive qualities in terms of their capacity to tune us into situations. There is an additional evolutionary psychiatric understanding of the adaptivity of “fearful anticipation and threat detection” as a naturally selected function (93). So, one option would be to assess changes in intuition in PPD by treating intuition as a predictive system that is either well-functioning or dysfunctional. What would have been or still can be “lifesaving” in an unsafe environment, namely, to always have our “guard up”, can also be seen as maladaptive under safe environmental circumstances, as in the case of prediction of threats in PPD. The adaptive role of intuition might be assessed in terms of *how flexible* one can adjust to environments in terms of personal goal attainment. There also might be living conditions in which one is primed to develop an attentional bias according to which anticipated threats can be (successfully) warded off. Without wanting to dive deeper into the evolutionary perspective on the etiological functional explanations of the predictive mechanisms [for a general view, see: Wakefield (94, 95), Wakefield et al. (96), Jacobs (97)], this explains at least *why* we have such a predictive capacity and adds a view to a mere heuristically perspective on the “adaptivity” of predictive capacities. The paranoid sensing of “threats” and anticipation of harm might then be understood as a dysfunction of the predictive system, which then, however, must integrate other neurobiological levels of explanation, such as amygdala hyperactivation [which has been explored in the case of schizophrenia, e.g., Pinkham et al. (98)] as occurring with an activation of the threat system in anxiety-related processes of paranoid experience. The “hyper-alert-mode” can be either assessed as biostatistically deviant or as an impairment of naturally selected functions. Alternatively, there is no such dysfunction of the mechanisms of the predictive system, as the respective mechanism functions exactly the way it has been naturally selected for. In the latter case, maladaptivity can be suggested solely with respect to the “harmful” impairments it might create in recent environments. It seems that predicting mostly threatening things happening can be assessed under most environmental conditions as rather socially maladaptive, predominantly as people with PPD might suffer from it, because other people can also become non-trivially harmed. Others may refrain from (further) support or from trying to reason with people with PPD. This furthermore illustrates how the biases become worse with difficult social experiences, such as facing obstacles of communicating paranoid “content” to other people, even when talking to medical professionals [cf. (99, 100)]. So, what must be considered is the role of environments regarding the onset and maintenance of paranoid disorders, particularly in hindering how people familiarize themselves with predicted threats, which is part of their adaptive potential.
(3) Third, it should be stressed that intuition is an *intersubjective enterprise*: we can share an experiential horizon with others as they are part of our “predictive” space. Individual oikeiôsis necessarily presupposes social recognition relations. From a conceptual and psychological view, it is also clear that an intuitive-sensing that *registers a lack of or disruption of familiarity, trust, and belonging* would not even be experientially possible without the important primordial (inter-affective) experiences of recognition relations that have matured the corresponding neural systems of our brains (101). It has been emphasized that *basic trust* and *knowing-how*, i.e., the acquisition of certain skills, are acquired through relation to others, which ideally are impregnated by affective resonance (102). I would like to stress that this is basically an *intuitive* process that allows one to experience trustful relatedness in the modes of an immediate, automatic, and experimental interaction with others. It has already been suggested that oikeiôsis is “neither as a purely individual nor a reason-driven process” [Fuchs (27, 28), p. 107, transl. KJ], but this “bodily mediated being-in-the-world”, which “originally develops as an inter-bodily being-with-others” [Fuchs (28), Ibid. transl. KJA], should be extended with respect to theories of an intuitive-sensing. Intuitive processing is inextricably intertwined with social *re*cognition, which implies the “ontogenetic and conceptual priority of the other” (103). This stresses how we bond and are primed by social interactions. Following this, the influence of distorted social recognition relations can be highlighted for their explanatory role in “distortions” of basic trust, feelings of familiarity, and belonging. The intersubjective-induced changes of the predictive system in PPD can be explained, for instance, in terms of developmental traumata/traumatic experiences. The onset and maintenance of paranoid oikeiôsis can be caused by relations that show a lack of recognition or are based on false recognition [cf. (104–107)] or manipulative environments [e.g., (108)] and are the roots of an erosion of basic trust. What has to be mentioned is that for PPD, the experiences of being manipulated, controlled, or betrayed can be suggested as an impairing factor for *developing a stable intuitive sense of self, others, and the world* ((109), 118ff), because if one constantly has to question the validity or reality of one's experiences, this can lead to constantly doubting the realness of one's subjective experiences and to anticipate subjection to threats or shaming (110). This has been a causal explanation in psychodynamic theory for the risk of developing PPD and paranoid psychosis (111, 112).

To sum up: Paranoid oikeiôsis involves a “crack” in what Karl Jaspers would have called the “shell” or “housing” [germ. *Gehäuse* cf. Jaspers (113), p. 281]. In more than one understanding of the following quote, paranoid experience is “[t]he conscious experience of borderline situations, which were previously concealed by the solid shell of objectively *self-evident* forms of life, world views, beliefs, and the movement of the boundless reflection, of the dialectical, began a process that brings the *previously self-evident housing to dissolution*.(...) [J]ust now it becomes more or less clear what the enclosure is, and this [is] experienced as *binding, restricting or as doubtful, without possessing the power to hold”* [Jaspers (113) transl. and italics KJA]. Often, these changes of oikeiôsis are so serious that they must be worked through therapeutically, which is now addressed in a final step.

## 3 The “oikeiôsis” therapy of paranoia

An “oikeiôtic” approach to PPD treatment stresses a holistic view of paranoid situatedness with respect to the impact environmental factors have on both pathogenesis *and* salutogenesis. The activation of resilience factors for an existential re-situating in terms of familiarity, trust, and social belonging is the main construction site of PPD treatment. This is already informed by the fact that PPD may come with diminished *illness* insight [for *insightfulness* in paranoia and the effects on internalization of stigmatization see: Valiente et al. (114)] and with lower treatment compliance. The therapeutic space must become “familiarized” as a place where the distrust and fears of being manipulated or not taken seriously by the therapist can become thematized.

There are (mixed) treatment methods—such as metacognitive interpersonal therapy (115), remediation-therapeutic approaches to train cognitive skills [as is indicated in schizophrenia, e.g., Wykes et al. (116)], cognitive analytic therapy [e.g., (117)], or different methods of introspection-based procedures in its refinements for PPD—to actively break the rigidity of perception, thought, and action patterns that often have a strong socially restrictive quality [such as loneliness, e.g., Bell et al. (118)]. The implementation of introspection-based methods for the treatment of paranoid experience might appear extra challenging since the client's “inner voice” holds persecutory beliefs and “feels under attack by a hostile other”, which is seen to influence the course of psychotherapy (119) and can limit the positive effects of therapy *broadly* and introspective work *specifically*. This is why therapy must be assessed for the risk of exacerbation and adjusted for each individual therapeutic case. Currently, however, the evidence for *mindfulness-based interventions* (MBI) in the treatment of PPD is increasing, especially to the extent that it has been shown to be effective for the treatment of PPD: it “appears to be adaptable to the unique features of different types of personality disorders” as cross-sectional, longitudinal, and experiential analysis suggests (120). Although the authors stress that further empirical research is required to reveal the effectiveness of mindfulness as a skill component and to identify the underlying mechanisms that cause therapeutic change, other studies also showed promising results, even in the treatment of distressing paranoid psychosis, which suggests that implementation of mindfulness training can impact cognition and affect specifically associated with paranoid beliefs [cf. (121, 122)]. Self-acquisition—as the meaning of oikeiôsis hints through—includes developing a self-reflexive understanding of one's (potentially misled, qua-biased) intuitive sense of self, others, and the world. The idea is that new experiences with others could be archived in the direction of establishing a sense of trust, familiarity, and security in aiming toward *an alteration of the relation to paranoid symptoms* in the first place and thematizing the intuiting of threats as a source of harmful impairments, particularly as being problematic in social interaction. The initial step to significantly reduce threatening experiences in the therapeutic setting might be to train to regulate intuitive irritations and develop the skills for insight into patients' biased intuitions about self, others, and the world. Mindfulness-based techniques might be integrated (in some) therapeutic models and may form the more “bodily-corporeal” access to stress reduction and insight, while acquiring insight in terms of an *understanding* of one's altered (intuitive) wiring toward others and the world may proceed in the method that Karl Jaspers has termed *genetic understanding* with his theory of *Existential Communication*. For Jaspers, psychotherapy is committed to modes of thinking and speaking that appeal to human freedom, which always *include* a critique of “*a misunderstood and misused freedom*” [cf. (123)] as the central problems of diagnostic and therapeutic communication. Jaspers has emphasized: “*By understanding I do not cause, but appeal to freedom. Through causal explaining I become able to intervene to a certain extent rationally calculable in the events in the sense of desired goals. But if I confuse the comprehensibility of meaning in the space of freedom and the causal explicability, I touch freedom. Then I treat it like an object, as if it were recognizably there, whereby I degrade it. And for this I miss causal possibilities that really exist”* [Jaspers (124) 221–230; 222; transl. KJA].

The tasks of *understanding psychology* include not only *static understanding*, i.e., a logically comprehensible description of the individual facts of the soul's life but also *genetic understanding*. While genetic understanding refers to the *subjective grasping of inner connections of meaning*, and thus to *understanding*—also in the sense of empathy—whereby it can also be distinguished, as it were, from the rational understanding of a sober logic of understanding, *explaining* is the objective pointing out of external connections according to the principle of causality [cf. Jaspers (125), *ibid*. chap. I/§ 3]. Where an intervention of the physician becomes justifiable as a causal explanation in recourse to the dimension of scientific knowledge, the conception of understanding—especially the concept of genetic understanding—immediately points to the practical realm of “*awakening thought, which brings to consciousness in us what we actually want*” [Jaspers (124), ibid, 304]. Thus, what matters in the therapeutic situation is to do justice to what can only be *understood* and becomes impossible in the attempt to objectify it. Transcending paranoid experiences in the communication of (losses of) freedom—especially under psychotherapeutic conditions—seeks to address a person in her life situation (oikeiôsis) about her destiny for freedom, which presupposes the “*belief in the value of the existent as freedom and its constitutive opening up to being and other existence*” [cf. (126)], which already points to the concept of insight [as self-illumination, cf. Jaspers (56), *ibid*. 35] that resonates with a mindfulness approach, as also for Jaspers an *acceptance* of one's existential situation is the decisive step in treatment. Acceptance follows the shock of realizing that one needs therapeutic help (the “crack”) and is followed by reminders that one's own destiny to freedom lies in facing this “crack”, as freedom echoes not only a “freedom from” but a “freedom toward”: maybe toward others will over time intuitively become felt again as less threatening. However, it must in all methodological modesty finally be stated regarding the challenges of treating PPD that an expectation of salvation in the sense of self-illumination (insight) always remains determined by the individual limits of autonomous self-contemplation. Thus, therapy “cannot *replace what life itself alone can bring*” [Jaspers (57), *ibid*. 35; italics in original].

## 4 Conclusion

I have examined PPD as a specific type of *oikeiôsis* and have suggested two readings of altered oikeiôsis: while the first appeals to the idea of “distorted” oikeiôsis in emphasizing how people feel restricted in their ability to ‘feel at home' and experience trustful relatedness, the second reading stresses the idea of “becoming situated and familiar” within a paranoid reality. With respect to the intuitive view on oikeiôsis, the procedural dynamics of the pre-reflective and (self-) reflexive spheres of evaluation have been sketched with respect to the role of (altered) intuition in PPD: Changes in intuitive processes are inextricably intertwined with attentional and interpretational biases, while conceptually both are bidirectionally related, thus stabilizing each other. Considering the main symptoms of PPD as an alteration of intuitive processing might provide an alternative view and is supported by models that stress the interface potential of cognitive science, statistical analysis, and philosophical phenomenology in future research on personality disorder. This more holistic understanding can be reflected by considering a combination of different therapeutic strategies, such as integrating introspection-based approaches in the treatment of PPD. I have finally emphasized a therapeutic notion of understanding that aims to induce a change in the relationship between clients and their symptoms. This *is* a change of oikeiôsis and ideally appeals to freedom in light of familiarization and trust.

## Author contributions

KJ: Writing–original draft.

## Footnotes

^1^Many thanks to reviewer 1 for the thoughtful comment on this less recognized connotation that can be discerned from the term itself.
